# The association between the Healthy Eating Index (HEI-2015) score and body composition among Iranian soccer players and referees: a cross-sectional study

**DOI:** 10.1017/jns.2022.49

**Published:** 2022-07-11

**Authors:** Mohammad Beba, Tohid Seif-Barghi, Sakineh Shab-Bidar, Habib Yarizadeh, Aliyu Jibril Tijani, Cain C. T. Clark, Kurosh Djafarian

**Affiliations:** 1 Department of Clinical Nutrition, School of Nutrition Sciences and Dietetics, Tehran University of Medical Sciences, Tehran 1416643931, Iran; 2 Department of Sports and Exercise Medicine, School of Medicine, Tehran University of Medical Sciences, Tehran 1417653911, Iran; 3 Department of Community Nutrition, School of Nutrition Sciences and Dietetics, Tehran University of Medical Sciences, Tehran 1416643931, Iran; 4 Center for Intelligent Healthcare, Coventry University, Coventry CV15FB, UK

**Keywords:** Body composition, Football, Healthy Eating Index (HEI-2015), Soccer referees, Soccer, BMC, bone mineral content, BMI, body mass index, HEI, Healthy Eating Index, PBF, percent body fat, PMM, percent muscle mass, WHR, waist-to-hip ratio, WHtR, waist-to-height ratio

## Abstract

For an optimal performance, soccer players and referees need to consume a high-quality diet. The Healthy Eating Index (HEI) is a tool that can estimate diet quality and has been shown to be associated with body composition. The aims of the present study were first to determine the HEI-2015 score of the diets consumed by athletes and second its association with different body composition parameters of athletes. We conducted a cross-sectional study on 198 soccer players and referees. Dietary intakes were recorded using a validated food frequency questionnaire (FFQ), and HEI scores were calculated. Body composition parameters were measured using the bioelectrical impedance analysis. The mean score for the HEI-2015 was 65⋅04. A multiple linear regression model showed significant associations of the HEI-2015 score with percent body fat (PBF), percent muscle mass (PMM), waist-to-hip ratio (WHR) and waist-to-height ratio (WHtR) in male soccer players aged <18 years, body mass index (BMI) in male soccer players aged ≥18 years and BMI and waist-to-height ratio (WHtR) in male soccer referees after adjustment for covariates (*P* < 0⋅05). The mean overall score for the HEI-2015 shows that Iranian soccer players and referees have an acceptable quality of diet. We also found significant associations between the HEI-2015 score and different body composition parameters in male soccer players and referees but we did not find any significant association in female athletes (*P* > 0⋅05).

## Introduction

Soccer is one of the most prevalent sports globally, with over 200 million people followers internationally^(1,2)^. While the soccer players play the most influential role in this sport^(3,4)^, the soccer referees have a critical role in the modern era^(5–8)^. The physical activity level of soccer referees during a match has been estimated to be around 10–12 km, with 4–18 % of this match-distance covered at speeds faster than 13–15 km/h^(9)^, which is comparable to what is observed in midfield players^(4,10)^, comparably, assistant referees cover between 5⋅8 and 7⋅3 km, varying according to the competition level^(11,12)^.

To sustain and improve the performance, soccer players and referees are advised to consume a high-quality diet, to help maintain an excellent age-related body composition. Optimal exercise performance, among many other factors, depends on body composition which may have an effect on athlete's strength, agility and appearance^(13)^. Therefore, a healthy diet with optimal calories, macro- and micronutrients is crucial for the performance of soccer players and referees during training and matches^(14–17)^.

Diet quality is often evaluated using different a priori indexes. Indeed, one of the most popular indexes is the Healthy Eating Index (HEI), which is based on aspects of the Dietary Guidelines for Americans (DGA)^(18)^, and indicates overall diet characteristics. Accumulating evidence shows that the HEI may be predictive of the risk of various health outcomes. Indeed, adherence to a healthy diet is an important strategy for the regulation of various biological processes associated with cardiovascular disease risk and body composition^(19)^. Several studies have been published on the association of single nutrients or energy and macronutrient intake and the performance of athletic populations^(20–22)^. Although several studies investigated the association between the HEI and body composition among children^(23,24)^ and healthy non-athletic adults^(25–28)^, to the best of our knowledge, no single study has investigated the association of the HEI-2015 score with body composition among both male and female soccer players and referees before. Different genetics of Iranians, Iranian traditional dishes and differences in their cooking methods and also higher physical activity level and nutritional knowledge among athletic *v*. non-athletic populations^(29–31)^ are among key determinants that can affect body composition and distinguish athletic and non-athletic populations and these differences clarify the novelty of the present study. Thus, the aims of the present study were first to determine the HEI-2015 score of the diets consumed by athletes and second its association with different body composition parameters of soccer players and referees.

## Materials and methods

### Study population and design

This was a cross-sectional study conducted on 198 elite (11 males and 23 females) and sub-elite (12 males and 24 females) soccer players and referees (89 males and 39 females) in Iran, during the early stages of the 2019–20 competitive season. Sub-elite soccer players (the national under-18 soccer players) were recruited for the present study. Elite soccer referees and assistant referees from all divisions, under the directive of the Soccer Federation of Iran, were also recruited for the present study. Data included participants’ demographics (age, gender and education), physical activity, medical history, anthropometric measurements and dietary assessment were assessed via a face-to-face interview with participants of the study. This study was conducted according to the guidelines laid down in the Declaration of Helsinki and all procedures involving human subjects were approved by the Tehran University of Medical Sciences Ethics Committee (Ethics number: IR.TUMS.VCR.REC.1398.729) and subjects were all given verbal and written communication about the study before signing an informed consent form.

Convenience sampling was used for the present study, and all players and referees who agreed to participate in the present study were included in the study. We used Brooke L. Devlin study to calculate the required sample size^(32)^. Fat-free mass (FFM) estimated the largest sample size among the other variables. Therefore, we calculated the power of the study based on this variable. The sample size required for the present study was estimated at 155 people but due to over- or under-reporting by some people and the possibility of losing some information, the sample size of 198 people was used. Collection of all information about anthropometric indices, demographic and lifestyle factors including dietary intakes and physical activities were done at the Medical Committee of the Islamic Republic of Iran Soccer Federation.

### Assessment of dietary intakes

Dietary data were collected using a 147-item semi-quantitative food frequency questionnaire (FFQ), which was validated in the Tehran Lipid and Glucose Study (TLGS). The validity and reliability of this 147-item semi-quantitative questionnaire have been published elsewhere^(33)^. This questionnaire examines food intakes of the past year; subsequently, dietary intake data were then entered into Nutritionist IV software modified for Iranian foods to estimate nutrient intake composition. Average energy, macro- and micronutrient intakes were also obtained.

### The HEI calculation

We used household measurements to calculate the score of each item listed in the HEI-2015. The HEI-2015 comprises 13 items in two main categories: adequacy and moderation, with a total score of 100. The first includes nine items, namely total and whole fruits, greens and beans, total vegetables, whole grains, dairy, total protein foods, seafood and plant proteins and fatty acids (polyunsaturated fatty acids (PUFA) + monounsaturated fatty acids (MUFA))/(saturated fatty acids (SFA)). The moderation category has four items, namely refined grains, sodium, added sugar and saturated fats. For whole fruits, total fruits, all vegetables, beans, peas, dairy products, total protein, seafood, plant proteins, whole grains, refined grains, and sodium, the HEI scoring standard is energy-adjusted (per 1000 kcal). Saturated fats and added sugar are calculated as a percentage of total calorie intake. The maximum point value is between 5 and 10 for each item. In the adequacy category, higher consumption leads to a higher score. If no food from one component is used, the component is given zero; if the recommended quantity, or more, is consumed, the maximum point value is obtained. In the moderation category, the maximum point value is attained if the recommended quantity or less is consumed (Supplementary Table S1).

### Assessment of body composition

Body composition was measured using the InBody 570 (InBody Co., Ltd. in Seoul, Korea), and analysed to quantify fat mass, percent body fat (PBF), lean mass, percent muscle mass (PMM) and bone mineral content (BMC). Calibration took place as per manufacturer guidelines. Participants’ measurements were conducted after an overnight fast and rest, without exercising on the morning of the scan. Participants were required to empty their bladder before each scan, and to wear minimal clothing. Athletes were advised not to consume caffeinated beverages at least 4 h before and drink at least two to four glasses of water 2 h before scanning. Scans were automatically analysed by the software. Body weight was measured with subjects in light clothing, upshot, using a digital scale (Seca 808, Germany) to the nearest 0⋅1 kg, while height was assessed using a wall-mounted stadiometer (Seca, Germany) to the nearest 0⋅1 cm. Body mass index (BMI) was calculated by dividing weight (kg) by height (m^2^). Waist circumference (WC) was measured at the midpoint of the lowest rib and iliac crest at the end of expiration using a measuring tape to the nearest 0⋅1 cm. Hip circumference was measured at the widest point over the buttocks using a measuring tape to the nearest 0⋅1 cm. Waist-to-hip ratio (WHR) was obtained by dividing the WC by the hip circumference.

### Assessment of physical activity

Information about physical activity was collected using a 7-item (short form) International Physical Activity Questionnaire (IPAQ). The validity and reliability of this questionnaire have been described and confirmed elsewhere^(34)^. This questionnaire asks participants about all types of physical activity that were done in the preceding 7 d. Individuals were divided into three groups in terms of physical activity:

1. Low activity: This group does not meet any of the criteria for subsequent groups.
2. Average: Having any type of physical activity (light, moderate or heavy) for 5 d or more in a week to meet 600 MET/min/week.
3. High activity: Having any type of physical activity (light, moderate or heavy) for 7 d in a week to meet 3000 MET/min/week.

### Statistical methods

The statistical package for social sciences (SPSS) version 25.0 (Chicago, IL, USA) for Windows was used for all statistical analyses and statistical significance was set at *P* < 0⋅05. Descriptive statistics (frequencies, cross-tabulation and *χ*^2^ value) were used to describe the main features of the data. Participants’ general characteristics were compared across tertiles of the HEI-2015 using analysis of variance (ANOVA) for continuous variables. Mean dietary intakes were compared across tertiles of the HEI-2015 scores using a general linear model, adjusted for sex, age (years, continuous) and physical activity level. Correlation statistics were used to discern the associations between each component of the HEI-2015 with the measures of body composition. To identify associations between the HEI-2015 scores with body composition (BMI, PBF, PMM, WHR, waist-to-height ratio (WHtR) and BMC), multivariate regression models were created, with adjustment for confounding variables.

All variables were tested for normality via the Kolmogorov–Smirnov statistic and visual assessment of histograms, and appropriate statistical tests were subsequently conducted. Data are presented as percentages, mean scores and standard deviations.

## Results

The general characteristics of the study participants across the tertiles of the HEI-2015 are shown in Table 1. A total of 198 volunteers participated in the present study, of which 112 (56⋅6 %) were males and 86 (43⋅4 %) were females. The mean age of participants was 29⋅36 ± 8⋅1 years, of that 36 (18⋅2 %) were aged under 18 years and 162 (81⋅8 %) were over 18 years of age. Of all participants, 70 (35⋅4 %) were soccer players and 128 (64⋅4 %) were soccer referees. There were no significant differences in the mean and frequency of other characteristics (*P* > 0⋅005). Mean of physical activity was 3002⋅62 ± 1839⋅55 MET/min/week, and, according to this, 144 (72⋅4 %) were moderately physical activity, and 54 (27⋅3 %) followed a high physical activity lifestyle.

**Table 1.** General characteristics of participants across tertiles of the Healthy Eating Index (HEI-2015)

	Healthy Eating Index								*P*-value
	Tertile 1 (*N* = 69)		Tertile 2 (*N* = 65)		Tertile 3 (*N* = 64)		Total		
	Mean	sd	Mean	sd	Mean	sd	Mean	sd	
Range	<61		62–68		>69				
Mean score^a^	56⋅47	3⋅89	65⋅13	1⋅98	74⋅17	4⋅49	65⋅04	8⋅1	**<0**⋅**001**
Age (years)^a^	27⋅74	8⋅10	29⋅25	8⋅68	31⋅22	8⋅58	29⋅36	8⋅10	0⋅06
<18	15 (21⋅7 %)		12 (18⋅5 %)		9 (14⋅1 %)		36 (18⋅2 %)		
≥18	54 (78⋅3 %)		53 (81⋅5 %)		55 (85⋅9 %)		162 (81⋅8 %)		
Gender^b^									
Male	41 (59⋅4 %)		38 (58⋅5 %)		33 (51⋅6 %)		112 (56⋅6 %)		0⋅61
Female	28 (40⋅6 %)		27 (41⋅5 %)		31 (48⋅4 %)		86 (43⋅4 %)		
Post^b^									
Soccer players	28 (40⋅6 %)		25 (38⋅5 %)		17 (26⋅6 %)		70 (35⋅4 %)		0⋅19
Soccer referees	41 (59⋅4 %)		40 (61⋅5 %)		47 (73⋅4 %)		128 (64⋅4 %)		
Physical activity^b^									
Moderate	54 (78⋅3 %)		47 (72⋅3 %)		43 (67⋅2 %)		144 (72⋅4 %)		0⋅35
High	15 (21⋅7 %)		18 (27⋅7 %)		21 (32⋅8 %)		54 (27⋅3 %)		
Physical activity^a^									
MET/min/week	2759⋅72	1575⋅45	2909⋅33	1449⋅92	3359⋅24	2358⋅83	3002⋅62	1839⋅55	0⋅15
Height (cm)^a^	172⋅94	7⋅88	173⋅43	8⋅90	171⋅51	8⋅53	172⋅63	8⋅43	0⋅407
Weight (kg)^a^	66⋅13	9⋅44	67⋅88	9⋅91	67⋅94	9⋅79	67⋅29	9⋅70	0⋅47
BMI (kg/m^2^)^a^	22⋅20	2⋅20	22⋅23	2⋅01	22⋅96	1⋅79	22⋅45	2⋅03	0⋅054
BFM (kg)^a^	12⋅10	4⋅19	12⋅40	3⋅28	13⋅59	3⋅67	12⋅68	3⋅78	0⋅057
PBF (%)^a^	18⋅49	6⋅53	18⋅42	4⋅68	20⋅28	5⋅71	19⋅05	5⋅74	0⋅11
FFM (kg)^a^	54⋅02	9⋅47	55⋅47	9⋅35	54⋅35	9⋅85	54⋅60	9⋅53	0⋅66
PMM (%)^a^	81⋅50	6⋅53	81⋅57	4⋅68	79⋅71	5⋅71	80⋅94	5⋅74	0⋅11
WHR^a^	0⋅82	0⋅04	0⋅81	0⋅03	0⋅82	0⋅03	0⋅81	0⋅03	0⋅85
WHtR^a^	0⋅45	0⋅03	0⋅45	0⋅02	0⋅46	0⋅02	0⋅45	0⋅03	0⋅13
BMC (kg)^a^	3⋅11	0⋅53	3⋅22	0⋅51	3⋅18	0⋅54	3⋅17	0⋅53	0⋅46

ANOVA, analysis of variance; BMI, body mass index; BFM, body fat mass; PBF, percent body fat; FFM, fat-free mass; PMM, percent muscle mass; WHR, waist-to-hip ratio; WHtR, waist-to-height ratio; BMC, bone mineral content.

a Values are expressed as mean values and standard deviations, by using one-way ANOVA.

b Values are reported as total and percentage, by using cross-tabulation and *χ*^2^ test.

*P*-value is considered significant at <0⋅05.

Bold values are statistically significant.

Scores for each component and the final score for the HEI-2015 are shown in Table 2. The mean final score for the HEI-2015 was 65⋅04 ± 8⋅1, and there was a significant difference between tertiles of the HEI-2015 score (*P* < 0⋅001). Furthermore all components, except greens and beans (*P* = 0⋅88), dairy (*P* = 0⋅93) and added sugar (*P* = 0⋅36) were significantly different across tertiles of the HEI-2015 score. Dietary intakes of total fruits, whole fruits and seafood and plant proteins had a significant difference between tertiles 1 and 2. Total vegetables, whole grains, fatty acids and sodium intakes indicated a significant difference between tertiles 1, 2 and 3. Total protein food intake had a significant difference between tertile 1 and tertile 3. Refined grains intakes showed a significant difference between tertile 2 and other tertiles. Saturated fat intakes had a significant difference between tertile 1 and other tertiles according to Tukey's test.

**Table 2.** Healthy Eating Index scores across tertiles of the Healthy Eating Index (HEI-2015)

Healthy Eating Index components	Healthy Eating Index								*P*-value
	Tertile 1 (*n* = 69)		Tertile 2 (*n* = 65)		Tertile 3 (*n* = 65)		Total		
	Mean	sd	Mean	sd	Mean	sd	Mean	sd	
Adequacy									
Total fruits	3·78	1⋅32	4·55	0·82	4·78	0·57	4·35	1·06	**<0**·**001**
Whole fruits	4·39	1·00	4·87	0·48	4·93	0·30	4·72	0·71	**<0**·**001**
Total vegetables	3·21	1·10	3·72	1·12	4·42	1·00	3·77	118	**<0**·**001**
Greens and beans	4·91	0·28	4·90	0·42	4·93	0·39	4·91	0·36	0·88
Whole grains	3·88	3·31	5·33	3·61	7·17	3·22	5·42	3·63	**<0**·**001**
Dairy	5·20	2·53	5·35	2·23	5·28	2·25	5·27	2·33	0·93
Total protein foods	3·71	1·00	4·01	1·12	4·18	0·88	3·96	1·02	**0**·**02**
Seafood and plant protein	1·26	0·47	1·61	0·74	1·81	0·95	1·55	0·77	**<0**·**001**
Fatty Acids (PUFA + MUFA/SFA)	3·04	2·69	4·18	2·60	5·70	2·65	4·27	2·85	**<0**·**001**
Moderation									
Refined grains	0·34	1·28	0·72	1·75	3·60	3·68	1·52	2·82	**<0**·**001**
Sodium	5·30	3·37	7·03	2·87	8·18	2·23	6·80	3·10	**<0**·**001**
Added sugar	10·0	0·00	9·98	0·12	10·0	0·00	9·99	0·71	0·36
Saturated fat	7·42	2·75	8·83	1·63	9·14	1·57	8·43	2·20	**<0**·**001**
Healthy Eating Index overall score	56·47	3·89	65·13	1·98	74·17	4·49	65·04	8·1	**<0**·**001**

ANOVA, analysis of variance; MUFA, monounsaturated fatty acids; PUFA, polyunsaturated fatty acids; SFA, saturated fatty acids.

Values are expressed as mean values and standard deviations. ANOVA analysis is used to compare means between tertiles of HEI-2015.

*P*-value is considered significant at <0·05.

Bold values are statistically significant.

The correlation between the HEI-2015 score and body composition parameters (BMI, PBF, PMM, WHR, WHtR and BMC) are shown in Table 3. Age, sex and physical activity were the most important covariates according to previous studies and differences observed in our own data. We found a significant correlation between the HEI-2015 score and PBF (*r* = 0⋅54, *P* = 0⋅03), PMM (*r* = −0⋅054, *P* = 0⋅03) and WHR (*r* = 0⋅51, *P* = 0⋅04) in under-18 male soccer players, BMI (*r* = 0⋅7, *P* = 0⋅008) in over-18 male soccer players and BMI (*r* = 0⋅331, *P* = 0⋅001) and WHtR (*r* = 0⋅247, *P* = 0⋅01) in male referees after adjusting for potential covariates, but we did not find any significant correlation between the HEI-2015 score and body composition parameters among female soccer players and referees.

**Table 3.** Association between the Healthy Eating Index (HEI-2015) scores and measures of body composition among different categories of athletes

Categories of athletes	BMI *r*^a^ (*P*)^b^	PBF *r*^a^ (*P*)^b^	PMM *r*^a^ (*P*)^b^	WHR *r*^a^ (*P*)^b^	WHtR *r*^a^ (*P*)^b^	BMC *r*^a^ (*P*)^b^
<18 male soccer players	−0⋅35 (0⋅45)	0⋅54 (0⋅03)*	−0⋅54 (0⋅03)*	0⋅51 (0⋅04)*	0⋅35 (0⋅13)	−0⋅04 (0⋅44)
≥18 male soccer players	0⋅7 (0⋅008)*	0⋅32 (0⋅16)	−0⋅32 (0⋅13)	0⋅25 (0⋅22)	0⋅508 (0⋅055)	0⋅31 (0⋅17)
<18 female soccer players	0⋅23 (0⋅14)	−0⋅107 (0⋅31)	0⋅107 (0⋅31)	−0⋅249 (0⋅12)	0⋅037 (0⋅43)	0⋅243 (0⋅12)
≥18 female soccer players	0⋅249 (0⋅12)	0⋅057 (0⋅39)	−0⋅057 (0⋅39)	0⋅115 (0⋅301)	0⋅115 (0⋅301)	0⋅239 (0⋅13)
Male soccer referees	0⋅331 (0⋅001)*	0⋅1 (0⋅17)	−0⋅1 (0⋅17)	0⋅083 (0⋅21)	0⋅247 (0⋅01)*	0⋅175 (0⋅05)
Female soccer referees	−0⋅064 (0⋅34)	−0⋅154 (0⋅17)	0⋅154 (0⋅17)	−0⋅240 (0⋅07)	−0⋅148 (0⋅18)	0⋅119 (0⋅23)

BMI, body mass index; PBF, percent body fat; PMM,  percent muscle mass; WHR, waist-to-hip ratio; WHtR, waist-to-height ratio; BMC, bone mineral content.

a ‘*r*’: Pearson correlation coefficient.

b *P*: significant value.

* Correlation is significant at the 0⋅05 level (two-tailed).

Bold values are statistically significant.

Results of the multiple linear regression model also indicate that there is a significant association between the HEI-2015 score and PBF (*P* = 0⋅004), PMM (*P* = 0⋅004), WHR (*P* = 0⋅03) and WHtR (*P* = 0⋅03) in under-18 male soccer players, BMI (*P* = 0⋅024) in over-18 male soccer players and BMI (*P* = 0⋅002) and WHtR (*P* = 0⋅03) in male soccer referees, but we did not find any significant association between the HEI-2015 score and body composition parameters among female soccer players and referees (Table 4).

**Table 4.** The association between the Healthy Eating Index (HEI-2015) scores with body composition with adjustment for confounders

Categories of athletes	Body composition parameters	HEI-2015		
		Unstandardised *β* coefficient	se	*P*-value
<18 male soccer players (*N* = 12)	BMI (kg/m^2^)	0·017	0·085	0·84
	PBF (%)	0·54	0·14	0·004*
	PMM (%)	−0·54	0·14	0·004*
	WHR	0·004	0·001	0·03*
	WHtR	0·002	0·001	0·03*
	BMC (kg)	−0·004	0·025	0·86
≥18 male soccer players (*N* = 11)	BMI (kg/m^2^)	0·18	0·067	0·024*
	PBF (%)	0·156	0·173	0·903
	PMM (%)	−0·156	0·173	0·39
	WHR	0·001	0·002	0·52
	WHtR	0·002	0·001	0·14
	BMC (kg)	0·029	0·023	0·24
<18 female soccer players (*N* = 24)	BMI (kg/m^2^)	0·051	0·042	0·24
	PBF (%)	−0·061	0·115	0·605
	PMM (%)	0·061	0·115	0·605
	WHR	−0·001	0·001	0·25
	WHtR	0·00	0·001	0·87
	BMC (kg)	0·012	0·010	0·24
≥18 female soccer players (*N* = 23)	BMI (kg/m^2^)	0·067	0·039	0·09
	PBF (%)	0·053	0·101	0·604
	PMM (%)	−0·053	0·101	0·604
	WHR	0·00	0·001	0·6
	WHtR	0·001	0·001	0·46
	BMC (kg)	0·007	0·007	0·302
Male soccer referees (*N* = 89)	BMI (kg/m^2^)	0·064	0·020	0·002*
	PBF (%)	0·030	0·047	0·53
	PMM (%)	−0·030	0·047	0·53
	WHR	0·00	0·00	0·61
	WHtR	0·001	0·00	0·03*
	BMC (kg)	0·009	0·005	0·08
Female soccer referees (*N* = 39)	BMI (kg/m^2^)	−0·028	0·042	0·49
	PBF (%)	−0·122	0·090	0·18
	PMM (%)	0·122	0·090	0·18
	WHR	−0·001	0·001	0·06
	WHtR	−0·001	0·001	0·21
	BMC (kg)	0·004	0·006	0·52

BMC, bone mineral content; BMI, body mass index; PBF, percent body fat; PMM, percent muscle mass; WHR, waist-to-hip ratio; WHtR, waist-to-height ratio.

A multiple linear regression model was created with adjustment for age, sex and physical activity level.

*P*-value is considered significant at <0·05.

* Bold values are statistically significant.

## Discussion

The mean value of the HEI-2015 score was 65⋅04 ± 8⋅1 and there was no significant difference between under-18 male soccer players, over-18 male soccer players, under-18 female soccer players, over-18 female soccer players, male soccer referees and female soccer referees (*P* = 0⋅32). The principal finding of the present study was that we found a significant association between the HEI-2015 score and different body composition parameters among male soccer players and referees but such an association was not found in female soccer players and referees. Interestingly, the association between the HEI-2015 score and body composition parameters was not consistent among different male populations. Based on some review studies, the age of the participants, differences in the tendency to consume certain groups of food and following certain dietary patterns at different ages, different dietary patterns in different regions, genetic predisposition, and the uncontrolled interventional variables are among potential causes which can affect body composition^(35)^. More importantly, during puberty, the main components of body composition (body fat mass, lean body mass and BMC) all increase, but these changes might be different between male and female individuals. During puberty, dramatic hormonal fluctuations as well as rapid growth in body size occur and are accompanied by marked changes in body composition. Furthermore, there are significant increases in the energy requirement of people in puberty age, associated with changes in body composition. These increases vary, are sex-dependent, and occur at an early stage of puberty as a result of an increase in physical capacities and energy expenditure^(36)^. These differences may partly explain the differences observed in the relationship between the HEI-2015 score and body composition among the different male population. Weinstein *et al.*^(37)^ conducted a study on adults above 17 years old, consisting of 16 467 people who participated in the NHANES study. The mean overall score for the HEI was 68⋅3, compared to a mean score of 65⋅04 in the present study. In another study, conducted in 2008, Ervin^(38)^ showed that the HEI overall score was 65⋅3 among men and 67⋅6 among women, in 3060 elderly people over 60 years old. Tande *et al.*^(39)^ reported that there was a significant association between the HEI score and abdominal obesity, where the mean overall score for the HEI was 63⋅72 among men and 65⋅1 among women. While the results of a study in 2012 showed that the mean score of the HEI-2005 in an elderly population was 54⋅08, highlighting an increase in whole grains, vegetables, beans and vegetable oils, and a decrease in their sodium intake is advisable^(40)^. Santos *et al.*^(41)^ conducted a cross-sectional study on twenty-one female soccer players, and reported the mean HEI-2010 overall score to be 54⋅6. Major carbohydrate sources in the HEI-2015 are total fruits, whole fruits, whole grains, refined grains and dairy products, indeed, mean score for total fruit and whole fruit were 4⋅35 and 4⋅72 out of 5, respectively, suggesting that our athletes were consuming adequate amounts of fruits. In contrast, consumption of whole grains and refined grains scores were 5⋅42 and 1⋅52, out of 10, respectively, which shows the population in our study were consuming insufficient amounts of whole grains and higher amounts of refined grains. Additionally, the dairy group had a score of 5⋅27 out of 10, which revealed that our study population did not consume enough dairy products. However, our population were considered to be consuming adequate protein-based foods (3⋅96 out of 5), although encouraging further increases in protein intake may be beneficial. According to the findings for seafood and plant protein (1⋅55 out of 5), it is evident that the athletes need to increase these sources of protein, especially seafoods, because they are also the most abundant sources of omega-3 fatty acids. PUFA + MUFA/SFA ratio was used to measure fatty acids score, and it was 4⋅27 out of 10, which is indicating that soccer players and referees did not achieve the right balance of consuming different types of fatty acids. The average sodium score was 6⋅81 out of 10, which may seem like an average score at first glance, but, importantly, there is no specific RDA for the amount of sodium needed in soccer, and it seems that the requirements for sodium in soccer players are more than normal people, largely due to increased training and perspiration.

The main finding of the present study was that a significant association between the HEI-2015 score and different parameters of body composition in male soccer players and referees was found; however, we were not able to find such an association among female soccer players or referees. While some studies have found non-significant associations between HEI scores with measures of body composition^(24,42)^, other studies have found significant associations^(25–28)^, highlighting the equivocality in the literature. As the present study was conducted during the early stage of the 2019–20 competitive season, the pre-season training or preparedness for the main season may, at least partly, explain broadly null findings between the HEI-2015 score and different measures of body composition in the present study. It has been reported that the body composition of soccer players is likely to alter during a competitive season as a result of training and competition stress^(43)^, habitual activity, and diet. Owen *et al.*^(44)^, in their study of the seasonal changes in body composition in elite European soccer players, observed a significant decrease in fat mass from the end of pre-season to the end of the season, compared with the start of pre-season. Furthermore, from the above study, similar significant changes were observed across the season for LBM, FFM and calf girth as a result of the training influence. Milanese *et al.*^(45)^ also observed that whole-body fat mass and PBF significantly decreased at mid-season and end-season, whereas fat-free skeletal tissue mass (FFSTM) significantly increased at mid-season and end of the season. Contrasting observations, however, have been reported by Clark *et al.*^(46)^, who found no significant changes in body composition within a squad of professional male soccer players across a season.

Our study has inherent strengths and limitations. Indeed, a FFQ which has been validated for the Iranian population was used to assess participants’ usual dietary intake. Also, all measurements and interviews were conducted by trained personnel. The results of the present study are generalisable to both males and females, and, to the best of our knowledge, this is the first study to have calculated the HEI-2015 score among Iranian soccer players and referees. However, we must acknowledge limitations that need to be addressed. First, the cross-sectional nature of the present study does not permit the assessment of causality. Only a prospective study would provide a better understanding of the association between micronutrient adequacy and body composition. Another limitation of our study is that we used InBody to assess body composition, and not dual X-Ray absorptiometry (DXA), which is considered the gold standard method. However, InBody is also a validated and reliable method for the measurement of body composition in the adult population^(47)^.

## Conclusion

The mean overall score for the HEI-2015 was 65⋅04 out of 100, which shows that Iranian soccer players and referees have an acceptable quality of diet. We also found a significant association between the HEI-2015 overall score and different body composition parameters in male soccer players and referees but no such association was seen in female soccer players or referees.

## Supplementary material

For supplementary material accompanying this paper visit https://doi.org/10.1017/jns.2022.49. click here to view supplementary material
