# Supplementary material for: The association between the Healthy Eating Index (HEI-2015) score and body composition among Iranian soccer players and referees: a cross-sectional study
Source: J Nutr Sci. 2022 Jul 11;11:e57. doi: 10.1017/jns.2022.49 (PMC9274382; doi:10.1017/jns.2022.49)
Supplement: Supplementary file 1 [file S2048679022000490sup001.docx]

Supplementary Table 1

Healthy Eating Index 2015 components, point values and standards for scoring

| Components | Maximum points | Standard for maximum points | Standard for minimum score of zero |
| --- | --- | --- | --- |
| Adequacy | | | |
| Total fruits | 5 | >0.8 c equivalents/1000 kcal | No fruits |
| Whole fruits | 5 | >0.4 c equivalents/1000 kcal | No whole fruits |
| Total vegetables | 5 | >1.1 c equivalents/1000 kcal | No vegetables |
| Greens and beans | 5 | >0.2 c equivalents/1000 kcal | No dark green vegetables or beans and peas |
| Whole grains | 10 | >1.5 oz equivalents/1000 kcal | No whole grains |
| Dairy | 10 | >1.3 c equivalents/1000 kcal | No dairy |
| Total protein foods | 5 | >2.5 c equivalents/1000 kcal | No protein foods |
| Sea food and plant proteins | 5 | >0.8 c equivalents/1000 kcal | No sea food or plant proteins |
| Fatty acids | 10 | (PUFAs+MUFAs)/SFAs >2.5 | (PUFAs+MUFAs)/SFAs <1.2 |
| Moderation | | | |
| Refined grains | 10 | <1.8 oz equivalents/1000 kcal | >4.3 oz equivalents/1000 kcal |
| Sodium | 10 | <1.1gr/1000 kcal | >2 gr/1000 kcal |
| Added sugar | 10 | <6.5 % of energy | >26 % of energy |
| Saturated fats | 10 | <8 % of energy | >16 % of energy |

MUFAs= Mono Unsaturated Fatty Acids, PUFAs= Poly Unsaturated Fatty Acids, SFAs= Saturated Fatty Acids
